# Genetic Diversity, Population Structure, and Resistance to *Phytophthora capsici* of a Worldwide Collection of Eggplant Germplasm

**DOI:** 10.1371/journal.pone.0095930

**Published:** 2014-05-12

**Authors:** Rachel P. Naegele, Samantha Boyle, Lina M. Quesada-Ocampo, Mary K. Hausbeck

**Affiliations:** 1 Department of Plant and Microbial Sciences, Michigan State University, East Lansing, Michigan, United States of America; 2 Department of Plant Pathology, North Carolina State University, Raleigh, North Carolina, United States of America; Virginia Tech, United States of America

## Abstract

Eggplant (*Solanum melongena* L.) is an important solanaceous crop with high phenotypic diversity and moderate genotypic diversity. Ninety-nine genotypes of eggplant germplasm (species (*S. melongena*, *S. incanum*, *S. linnaeanum* and *S. gilo*), landraces and heirloom cultivars) from 32 countries and five continents were evaluated for genetic diversity, population structure, fruit shape, and disease resistance to Phytophthora fruit rot. Fruits from each line were measured for fruit shape and evaluated for resistance to two *Phytophthora capsici* isolates seven days post inoculation. Only one accession (PI 413784) was completely resistant to both isolates evaluated. Partial resistance to Phytophthora fruit rot was found in accessions from all four eggplant species evaluated in this study. Genetic diversity and population structure were assessed using 22 polymorphic simple sequence repeats (SSRs). The polymorphism information content (PIC) for the population was moderate (0.49) in the population. Genetic analyses using the program STRUCTURE indicated the existence of four genetic clusters within the eggplant collection. Population structure was detected when eggplant lines were grouped by species, continent of origin, country of origin, fruit shape and disease resistance.

## Introduction

Cultivated eggplant, *Solanum melongena* L., is a high-value vegetable commodity in Europe and Asia. China and India are the major producers with 27.7 and 11.9 million tons per year, respectively [2011 FAO]. Eggplant is the third most important solanaceous crop worldwide after potato and tomato, and fourth most important in the U.S. [2011 FAO]. In the U.S., eggplants are a minor crop grown for specialty markets with an approximate production of 62 thousand tons annually [2011 FAO].

Unlike most other cultivated solanaceous crops (tomatoes, peppers, and potatoes), eggplants are an Old World species. Domestication of the cultivated eggplant is thought to have occurred in Asia as early as 59 B.C. [1]–[3]. Since that time, it has been transported and cultivated around the world [4], [5]. Primary and secondary centers of diversity for eggplant are Asia and the Mediterranean basin in Europe, respectively. Studies have indicated that the progenitors of domesticated eggplant (*S. melongena*) originated in Africa and were derived from the closely related *Solanum incanum* (part of the eggplant complex) [6] and *Solanum linnaeanum* [5]. Both *S. incanum* and *S. linnaeanum* can form partially fertile hybrids with *S. melongena* making them potential sources for desirable traits such as abiotic and biotic disease resistance [7]–[9]. Wild relatives have traditionally been a good source of disease resistance for cultivated species that exhibit lower genetic diversity [10]–[15]. Domesticated heirloom varieties and landrace accessions may also harbor resistance, and are often more similar to modern cultivated varieties than wild species, making them a good source for desirable traits [16]–[17].

Multiple *Phytophthora* species are capable of causing disease symptoms on eggplants. Infected eggplants can display root and/or fruit symptoms. One causal agent of Phytophthora fruit rot is *Phytophthora capsici* L., an oomycete that affects multiple solanaceous species including eggplant, pepper, and tomato [18]–[22]. In the field, chemical management is expensive and provides limited protection against *Phytophthora capsici*-induced fruit rot in eggplant, which is the most common *Phytophthora*-induced symptom in eggplants [23]. Cultivated eggplants have some level of root rot resistance to moderately virulent isolates of *P. capsici* [20]. No cultivars, to date, have displayed any type of fruit resistance. Host resistance, an important part of a successful, sustainable management program, is not available for management of Phytophthora fruit rot in eggplants and currently, no known lines or cultivars are resistant to *P. capsici*. Partial fruit rot resistance to *P. capsici* has been identified in other solanaceous species such as peppers and tomatoes ([24], Granke et al. unpublished), but to our knowledge this has not been evaluated in eggplant.

In addition to disease resistance, fruit shape is an important attribute for each cultivar and many studies have been performed to identify the genetic basis of fruit shape in the Solanaceae [25]–[30]. Size, shape and color vary greatly between eggplant market classes, and it will be important to maintain this phenotypic diversity when incorporating disease resistance [4], [31]–[32]. This phenotypic diversity does not always translate to high levels of genetic diversity [4], [33]. Modern varieties of eggplant often have lower genetic diversity, and new traits are often bred into commercial varieties from landraces or wild relatives with higher genetic diversity [34]. The characterization of genetic diversity is important for maintenance and utilization of germplasm resources (wild, landrace, heirloom, breeding lines and cultivars), and the development of core collections [3], [35]. Genetic bottlenecks (domestication, selection of lines by market class, etc.) have limited the variability existing within cultivated lines [3].

Population structure analysis has recently been gaining popularity as a method to understand and visualize spatial and temporal differences between subpopulations [19], [36]–[40]. Information on population structure can provide insight about connections between phenotypic variation and the distribution of genetic diversity. Population structure should also be taken into account when testing and incorporating desirable traits. If population structure is present in materials being evaluated for association mapping, spurious associations may be found between a particular genotype and the trait of interest [31], [41]. Many studies have looked at the genetic diversity of eggplants within specific countries or regions, and a recent study compared genetic differentiation and structure in three centers of diversity; however, few studies have looked at diversity and population structure in a global collection of eggplants [2], [4], [31], [34], [42]–[46].

We evaluated the fruit shape index, Phytophthora fruit rot resistance, genetic diversity and population structure of a diverse collection of eggplant germplasm using 22 simple sequence repeats (SSRs). Our objectives were to evaluate a worldwide collection of eggplants for population structure and genetic diversity, and to determine if the population structure is associated with fruit shape or resistance to *Phytophthora capsici*. These results provide an initial basis for understanding the worldwide population structure of eggplants for breeding and conservation, and its relationship with disease resistance and fruit shape.

## Materials and Methods

### Permissions

No specific permits were required for the described field studies, which took place in an experimental field plot at Michigan State University (MSU). This field plot was used by the authors of this paper affiliated to the aforementioned institution (RN, SB, MK, LQ) for field trials to evaluate eggplant germplasm and cultivated species.

### Plant Material

Ninety-four accessions of eggplants (*S. melongena*), three accessions of *S. linnaeanum*, one accession of *S. gilo*, and one accession of *S. incanum* were obtained from the United States Department of Agriculture Germplasm Resource Information Network (ars-grin.usda.gov), Universidad de Technologia de Valencia, and the INRA (French National Institute for Agricultural Research) (Table 1). Accessions represented 32 countries on five different continents, and included primary and secondary centers of diversity. Seeds were sown into 72-cell trays containing a soilless peat mixture (Suremix Michigan Grower Products, Inc. Galesburg, MI) in a polyethylene greenhouse (MSU Horticulture Teaching and Research Center, Holt, MI). Eight weeks after sowing, seedlings were transplanted into a field at the MSU Plant Pathology Research Center (East Lansing, MI). Individual accessions were planted into single plots. Each individual line was established in 3 m long plot and 12 lines were planted per row. Within rows, plants were spaced 0.45 m apart. Rows were spaced 2.4 m apart, covered with black plastic mulch, and grown according to local practices. Immature eggplant fruits of marketable size were hand harvested throughout the growing season and brought to the lab for inoculation and evaluation.

**Table 1:** Eggplant germplasm used for the study of morphological and molecular variation.

Species	ID	Accession	Plant ID	Country	Source
*S. melongena*	101	C-S-16	-	Spain	UT Valencia
*S. melongena*	102	Grif 1276	46B	Thailand	USDA-GRIN
*S. melongena*	103	Grif 14182	New Orleans Market	U.S.	USDA-GRIN
*S. melongena*	104	Grif 14186	Hastings purple thornless	U.S.	USDA-GRIN
*S. melongena*	105	H15	-	Spain	UT Valencia
*S. melongena*	106	IVIA-371	-	Spain	UT Valencia
*S. melongena*	107	MM 108 bis	-	France	AVRDC
*S. melongena*	108	MM 114	Berengena larga negra	Spain	AVRDC
*S. melongena*	109	MM 1171	Large Santa Olalla	Spain	AVRDC
*S. melongena*	110	MM 1363	-	Costa Rica	AVRDC
*S. melongena*	111	MM 1364	-	Costa Rica	AVRDC
*S. melongena*	112	MM 1365	-	Guatemala	AVRDC
*S. melongena*	113	MM 141	Violette d'Avignon	France	AVRDC
*S. melongena*	114	MM 1750	Listada di Gandia	Spain	AVRDC
*S. melongena*	115	MM 346	Berengena redonda	Spain	AVRDC
*S. melongena*	116	MM 39	Noire de Chateaurenard	France	AVRDC
*S. melongena*	117	MM 522	Waimanolo long B1	U.S.	AVRDC
*S. melongena*	118	MM 56	Violette de Toulouse	France	AVRDC
*S. melongena*	119	MM 61	Zebrina	Spain	AVRDC
*S. melongena*	120	MM 64	Ronde de Valence	France	AVRDC
*S. melongena*	121	MM 69	Monstrueuse de New York	U.S.	AVRDC
*S. melongena*	122	MM 91	Black Beauty	U.S.	AVRDC
*S. melongena*	123	PI 102727	No. 202	Uzbekistan	USDA-GRIN
*S. melongena*	124	PI 105346	Lao Lai Hei Chieh	China	USDA-GRIN
*S. melongena*	125	PI 115505	Giant of Benares	India	USDA-GRIN
*S. melongena*	126	PI 140446	5917	Iran	USDA-GRIN
*S. melongena*	127	PI 140456	7015	Iran	USDA-GRIN
*S. melongena*	128	PI 141968	No. 1	China	USDA-GRIN
*S. melongena*	129	PI 143410	Badenjan	Iran	USDA-GRIN
*S. melongena*	130	PI 169641	1448	Turkey	USDA-GRIN
*S. melongena*	131	PI 169650	2259	Turkey	USDA-GRIN
*S. melongena*	132	PI 171851	6753	Turkey	USDA-GRIN
*S. melongena*	133	PI 175914	9043	Turkey	USDA-GRIN
*S. melongena*	134	PI 179500	9877	Iraq	USDA-GRIN
*S. melongena*	135	PI 179997	10598	India	USDA-GRIN
*S. melongena*	136	PI 181896	Aleppo 3	Syria	USDA-GRIN
*S. melongena*	137	PI 181963	Homs 21	Syria	USDA-GRIN
*S. melongena*	138	PI 193599	Long Violet	Ethiopia	USDA-GRIN
*S. melongena*	140	PI 199516	M 19	Greece	USDA-GRIN
*S. melongena*	141	PI 200881	-	Afghanistan	USDA-GRIN
*S. melongena*	142	PI 204731	-	Turkey	USDA-GRIN
*S. melongena*	143	PI 213193	M-57/29	Greece	USDA-GRIN
*S. melongena*	144	PI 217962	Banjal Bemba	Pakistan	USDA-GRIN
*S. melongena*	145	PI 223844	-	Philippines	USDA-GRIN
*S. melongena*	146	PI 230333	Kairyo-onaga	Japan	USDA-GRIN
*S. melongena*	147	PI 230334	Kitta Horyo	Japan	USDA-GRIN
*S. melongena*	148	PI 230335	Taiwan-naga	Japan	USDA-GRIN
*S. melongena*	149	PI 232078	Kopek	South Africa	USDA-GRIN
*S. melongena*	150	PI 232079	Mofale	South Africa	USDA-GRIN
*S. melongena*	151	PI 233916	-	El Salvador	USDA-GRIN
*S. melongena*	152	PI 234632	Early Round Purple	South Africa	USDA-GRIN
*S. melongena*	153	PI 241506	Badanjan	Iran	USDA-GRIN
*S. melongena*	154	PI 249570	Makhua Proh	Thailand	USDA-GRIN
*S. melongena*	155	PI 256077	No. 1	Afghanistan	USDA-GRIN
*S. melongena*	156	PI 263727	Rosita	Puerto Rico	USDA-GRIN
*S. melongena*	157	PI 267104	Cylinder A-132	Soviet	USDA-GRIN
*S. melongena*	158	PI 269600	423	Pakistan	USDA-GRIN
*S. melongena*	159	PI 276104	Motale	South Africa	USDA-GRIN
*S. melongena*	160	PI 286099	No. 62-46-2	U.S.	USDA-GRIN
*S. melongena*	161	PI 286100	No. 62-48-2	U.S.	USDA-GRIN
*S. melongena*	162	PI 290467	Lungi de Impant	Hungary	USDA-GRIN
*S. melongena*	163	PI 290469	Cu-e-da-juan	Hungary	USDA-GRIN
*S. melongena*	164	PI 304839	G2562	Brazil	USDA-GRIN
*S. melongena*	165	PI 320501	24	Canada	USDA-GRIN
*S. melongena*	166	PI 320504	28	Canada	USDA-GRIN
*S. melongena*	167	PI 320509	35	Canada	USDA-GRIN
*S. melongena*	168	PI 349612	Terongglatik	Indonesia	USDA-GRIN
*S. melongena*	169	PI 351129	Kurume Long	Japan	USDA-GRIN
*S. melongena*	170	PI 358232	Dolg	Macedonia	USDA-GRIN
*S. melongena*	171	PI 358242	Morska Pata	Macedonia	USDA-GRIN
*S. melongena*	172	PI 358244	Renski dolg	Macedonia	USDA-GRIN
*S. melongena*	173	PI 368822	Sredno Dolg	Macedonia	USDA-GRIN
*S. linnaeanum*	174	PI 388846	WL-74	Italy	USDA-GRIN
*S. linnaeanum*	175	PI 388847	WL-85	Italy	USDA-GRIN
*S. melongena*	176	PI 391646	Liu-ye-ch'ieh	China	USDA-GRIN
*S. melongena*	178	PI 413782	22–73	Cote D'Ivoire	USDA-GRIN
*S. melongena*	179	PI 413783	3–73	Burkina Faso	USDA-GRIN
*S. melongena*	180	PI 413784	13–73	Burkina Faso	USDA-GRIN
*S. melongena*	181	PI 419198	Tsu Yang	China	USDA-GRIN
*S. linnaeanum*	182	PI 420415	52	Colombia	USDA-GRIN
*S. gilo*	183	PI 441908	BGH 5008	Brazil	USDA-GRIN
*S. melongena*	184	PI 452122	Lunga Violetta di Romagna	Italy	USDA-GRIN
*S. melongena*	185	PI 452123	Tonda di Manfredonia	Italy	USDA-GRIN
*S. melongena*	186	PI 462370	Neznyj 36	Soviet	USDA-GRIN
*S. melongena*	187	PI 470273	-	Indonesia	USDA-GRIN
*S. melongena*	189	PI 478390	O 81	China	USDA-GRIN
*S. melongena*	190	PI 491192	Kemer	Turkey	USDA-GRIN
*S. incanum*	191	PI 500922	Chipusni	Zambia	USDA-GRIN
*S. melongena*	192	PI 560903	Six Leaves	China	USDA-GRIN
*S. melongena*	193	PI 561139	37	Kazakhstan	USDA-GRIN
*S. melongena*	194	PI 561140	36	Kazakhstan	USDA-GRIN
*S. melongena*	195	PI 593748	56A	Thailand	USDA-GRIN
*S. melongena*	196	PI 593806	171	Thailand	USDA-GRIN
*S. melongena*	198	PI 593885	314	Thailand	USDA-GRIN
*S. melongena*	199	PI 595220	Gator	United States	USDA-GRIN
*S. melongena*	200	PI 600912	Little fingers	U.S.	USDA-GRIN
*S. melongena*	201	PI 606714	Pompano market	U.S.	USDA-GRIN
*S. melongena*	202	PI 639121	Puerto Rican beauty	Puerto Rico	USDA-GRIN
*S. melongena*	203	PI 639122	Blackee	U.S.	USDA-GRIN

### Isolates

Two virulent *P*. *capsici* isolates, previously evaluated on pepper, tomato and eggplant, were selected from the long-term collection of Dr. Mary K. Hausbeck at MSU [18]–[20]. Isolates were characterized by host of origin, mefenoxam sensitivity [insensitive (I) or sensitive (S)] and mating type (A1 or A2). Isolate 12889 (pepper, I, A1) and isolate OP97 (cucumber, S, A1) were maintained on unclarified V8 agar at 25°C under constant light. Prior to inoculations, isolates were activated by inoculating and recovering each isolate from an individual pepper fruit to ensure virulence.

### Inoculation and Evaluation

For inoculation, a single 6 mm-diameter plug from an actively growing *P. capsici* isolate on V8 agar was placed, mycelium side down onto a non-wounded eggplant fruit surface-disinfested in 10% bleach for 5 min and rinsed with distilled water. Control eggplants were inoculated with a single 6 mm-diameter sterile plug of agar. Plugs were covered with a sterile microcentrifuge tube and affixed into place with petroleum jelly. Eggplants were placed into a humidity chamber consisting of an aluminum pan with a ring of moistened paper towel around the edge, covered with plastic wrap, sealed with tape and kept under constant light at room temperature (25°C). Three fruits (replicates) from each eggplant line were evaluated per isolate. The experiment was performed three times (runs). An experiment replicate included three fruits for each isolate of every eggplant accession evaluated in a completely randomized design (CRD) blocked by isolate. One line (PI 500922) was repeated only one time for a total of two experimental replicates of three fruit per isolate due to poor fruit set. Two control fruits were inoculated with a sterile plug of V8 agar for each line.

**Figure 1:**
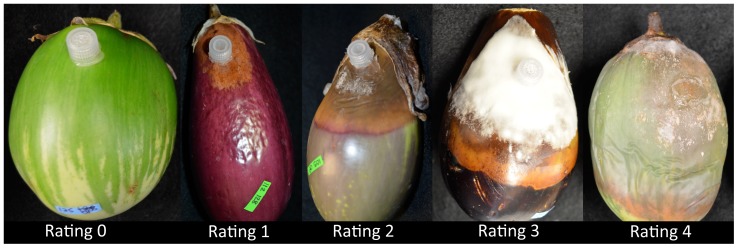
Eggplant Phytophthora fruit rot disease rating scale shown on various eggplant genotypes: 0 =  no visible symptoms, a rating of 1 = <25% symptomatic area, 25%>2<50%, 50%>3<75%, and a rating of 4≥75% symptomatic area of the fruit.

Eggplant fruits were evaluated for disease severity seven days after inoculation. Fruits were evaluated on the following progressive scale based on the percentage of symptomatic fruit surface to account for differences in fruit size: 0 =  no visible symptoms (resistant (R)), 1 = <25% of the fruit was symptomatic (moderately resistant (MR)), 2 = 25% to <50% (moderately susceptible (MS)), 3 = 50% to <75% (susceptible (S)), 4 = ≥75% symptomatic area (susceptible (S)) (Fig. 1). Visible mycelia growth was assessed as 0 =  absent, 1 =  present. *Phytophthora* isolations were performed on 10% of symptomatic fruits by peeling back the external layer of the fruit and plating three small portions of fruit tissue at the disease margin onto V8 agar plates amended with benomyl, ampicillin, PCNB, and mefenoxam to confirm the causal agent of the symptoms [47]. *Phytophthora capsici* was identified using morphological characteristics according to Waterhouse [48] and isolate mefenoxam sensitivity was confirmed by transferring the recovered isolates to V8 plates amended with 100 ppm mefenoxam according to Lamour and Hausbeck [47].

Ten immature fruits of marketable size collected from each line were measured for maximum length (cm) and maximum width (cm) using a hand caliper. Fruit shape was calculated as the ratio of maximum length to midpoint width for each line. Fruit shape ratios were rounded to the nearest whole number. Values between 0 and 1 were considered round, 2–3 were considered oval, 4–5 were semi-elongate and >5 were considered elongate.

### Phenotype Statistical Analyses

Mean values for disease ratings for each accession were estimated using the PROC MEANS function of SAS software v9.3 (SAS Institute, Cary, NC). Significant differences between disease values (ratings) for eggplant accessions and isolates were estimated using the PROC MIXED function of SAS software. Significant differences were detected between experiment runs and each run was analyzed separately using Fisher's LSD test (*P* = 0.05). Accession by isolate interactions were calculated using the ANOVA slice option of PROC MIXED when *P*≤0.05. Lines with a consistent disease mean value of ≥2 in each run of the experiment were considered susceptible, with a consistent mean value <2 were termed moderately susceptible, lines with a consistent mean value <1 were moderately resistant, and lines with a mean value  = 0 were resistant. Significant differences for pathogen growth were estimated using the PROC GLIMMIX function of SAS at *P* = 0.05.

Fruit shape significant differences between lines and countries were calculated using the PROC mixed function of SAS software v9.3. Countries represented by less than four accessions were excluded from analyses. Unequal sample sizes among countries were accounted using the Kenward-Rogers degrees of freedom option implemented in SAS software. Line mean values for fruit shape, length and width were calculated using the lsmeans statement of SAS software. Correlations between fruit shape parameters and disease susceptibility were estimated using the PROC CORR function of SAS. Disease susceptibility correlations were evaluated for each isolate and experimental replicate separately.

### Genetic Analyses

Genomic DNA was extracted from young green leaves of eggplants using the Nucleo Spin II DNA extraction kit (Machery-Nagel Germany, CAT#740770) according to the manufacturer's instructions. DNA was normalized to 5 ng/ µl using the NanoDrop ND 1000 spectrophotometer and NanoDrop 2.4.7c software (NanoDrop Technologies Inc., Wilmington, DE).

One hundred ninety-two primers from previously published SSR markers ([3], [49], solgenomics.org) or designed (Primer 3 http://primer3.sourceforge.net/) from putative Solanaceae defense-related genes (NCBI http://ncbi.nlm.nih.gov) were tested against a subset of the eggplant collection to identify polymorphic markers. Reactions were performed in 15 µl total volume and contained 1 µl DNA, and 0.15 µl GoTaq (Promega Corporation Madison, WI), 0.9 µl 25 µM MgCl_2_, 0.3 µl dNTPs, and 0.6 µl each of forward and reverse primers (Integrated DNA Technologies, Inc., Coralville, IA), with 8.45 µl ddH_2_O. PCR reactions were performed in a programmable thermal cycler (Eppendorf, Westbury, NY) using the program: initial denaturation, 94°C (3 min) followed by 35 cycles at 94°C (30 s), 60°C (30 s) and 72°C (1 min), with a final extension step of 10 min at 72°C. PCR products were analyzed by electrophoresis in 4% (wt/vol) agarose gel in 1× Tris-borate-EDTA buffer, stained with ethidium bromide (5 ug/ml) for visualization and compared to a 100-bp ladder (Invitrogen Life Technologies, Burlington, ON, Canada) to determine amplicon sizes. SSR markers identified as polymorphic in the population were used for genetic diversity, population structure and trait associations.

### Genetic Diversity and Population Structure

Genetic diversity was estimated using Powermarker v3.25 [50] and significance at each locus was determined with 1000 permutations using the Exact test; overall genetic diversity was estimated using the Mantel test as implemented in Powermarker. Genetic distance matrix values were calculated using Euclidean distance with the unweighted pair group method with arithmetic mean (UPGMA) and visualized in MEGA5 [50], [51].

Population structure of the germplasm was analyzed using STRUCTURE v2.3.4 [52]. Following preliminary analyses, burnin length, MCMC chain replication and lambda were selected to be 200,000, 500,000 and 1.52, respectively. Population number (k) was determined empirically by comparing posterior distribution likelihoods independently among 3 independent runs of K = 1 to 20 as described by Evanno et al. [53]. Data included 22 polymorphic SSRs and were analyzed using the admixture model and correlated allele frequencies without previous population information [52], [54]. Wright's subpopulation fixation index (Fst), the proportion of the total genetic variance within a subpopulation, significance between populations was determined using 1000 bootstrap replicates as implemented in Powermarker [55].

Visualization of the resulting Q (proportion of membership based on a 0 to 1 scale) of each accession into predefined categories (country, continent, species, disease susceptibility and fruit shape) was generated using the Population Sorting Tool (PST) in R [19], [56] (J.J. Morrice, unpublished). Individuals with Q ≥0.6 membership in a single subpopulation were labeled as such. Individuals with Q<0.6 membership in a single subpopulation were considered admixed. Significance of population structure predefined categories was estimated using the population differentiation test implemented in Powermarker. Significance at each locus and overall was determined using 1000 permutations. Countries represented by less than four individuals were excluded from analyses. Significance of pairwise Fst differentiation was based on 2.5% and 97.5% confidence intervals (*P* = 0.05) based off of 1000 bootstrap replications.

## Results

### Phytophthora capsici Disease Resistance and Fruit Shape

Significant differences between experimental runs indicated the effect of environmental variability during fruit growth and development on fruit disease resistance was high. In each repetition of the experiment, there were significant differences among plant accessions (*P*<0.0001). No significant differences in disease severity were found between isolates in any replicate of the experiment (*P* = 0.32, *P* = 0.43, and *P* = 0.43). The interaction between accessions and isolate was significant for each run (Run 1: *P* = 0.0008; Run 2: *P*<0.0001; and Run 3 *P*<0.0001) of the experiment. Differences in pathogen growth (absence/presence) and the interaction between pathogen growth and accession were not significant in any replicate (approximately *P* = 1.0 for each). The majority of the accessions were susceptible at 7 days post inoculation to isolates OP97 (89%) and 12889 (87%), respectively (Table 2). Symptoms included brown discoloration of the fruit and water soaking, with occasional external mycelial growth (Fig 1). Eggplant accession PI 413784 was the only line completely Rto both isolates tested. Susceptibility to one isolate did not always result in susceptibility to the other isolate. Lines PI 413782 and Grif 1276 were R (rating  = 0) to isolate OP97 and S or MS to isolate 12889. *S. melongena* lines, MM1365, PI 193599, PI 263727 and PI 419198 were MS (rating <2) to isolate OP97. Eggplant lines H15 and PI 441908 were R to isolate 12889 and S and MS to isolate OP97, respectively. Two of the *S. linnaeanum* lines, PI 388847 and PI 388846, and the *S. incanum* accession PI 500922 were R or MR to isolate 12889. Lines PI 181896, PI 233916, MM 56, and PI 413783 were MS to isolate 12889. *Phytophthora capsici* isolates were successfully recovered from diseased fruits and mefenoxam sensitivity was confirmed for each isolate (*data not shown*).

**Table 2:** Fruit shape parameters and mean disease ratings for each isolate.

		Mean^a^		Fruit^b^			
Species	Accession	12889	OP97	Ratio	Length	Width	Cluster^c^
*S. melongena*	C-S-16	S	S	5.59	4.12	22.98	1
*S. melongena*	Grif 1276	MS	R	1.14	4.30	4.92	2
*S. melongena*	Grif 14182	S	S	2.59	6.11	14.97	3
*S. melongena*	Grif 14186	S	S	1.75	7.83	13.48	1
*S. melongena*	H15	R	S	1.93	6.17	11.81	2
*S. melongena*	IVIA-371	S	S	2.08	7.26	14.98	4
*S. melongena*	MM 108 bis	S	S	5.23	4.21	21.52	1
*S. melongena*	MM 114	S	S	6.57	3.44	22.23	2
*S. melongena*	MM 1171	S	S	2.81	5.43	15.14	1
*S. melongena*	MM 1363	S	S	5.05	5.12	25.77	2
*S. melongena*	MM 1364	S	S	3.02	6.02	17.78	5
*S. melongena*	MM 1365	S	MS	1.87	7.88	14.55	2
*S. melongena*	MM 141	S	S	4.67	5.14	23.84	1
*S. melongena*	MM 1750	S	S	2.49	7.11	17.52	5
*S. melongena*	MM 346	S	S	1.31	8.73	11.35	2
*S. melongena*	MM 39	S	S	5.37	4.29	22.89	1
*S. melongena*	MM 522	S	S	8.03	3.27	26.14	1
*S. melongena*	MM 56	MS	S	2.42	6.83	16.33	2
*S. melongena*	MM 61	S	S	1.97	5.88	11.53	2
*S. melongena*	MM 64	S	S	1.16	8.65	9.99	2
*S. melongena*	MM 69	S	S	1.34	8.78	11.43	4
*S. melongena*	MM 91	S	S	1.92	7.49	14.19	4
*S. melongena*	PI 102727	S	S	2.44	6.10	14.76	4
*S. melongena*	PI 105346	S	S	1.17	8.94	10.33	4
*S. melongena*	PI 115505	S	S	1.72	6.57	11.27	2
*S. melongena*	PI 140446	S	S	1.77	7.14	12.55	2
*S. melongena*	PI 140456	S	S	3.48	6.31	21.85	5
*S. melongena*	PI 141968	S	S	4.46	4.52	19.91	2
*S. melongena*	PI 143410	S	S	1.35	7.94	10.68	5
*S. melongena*	PI 169641	S	S	3.78	5.19	19.34	4
*S. melongena*	PI 169650	S	S	4.66	4.22	19.20	4
*S. melongena*	PI 171851	S	S	4.31	4.14	17.78	4
*S. melongena*	PI 175914	S	S	2.92	4.83	14.08	5
*S. melongena*	PI 179500	S	S	3.64	4.41	16.01	2
*S. melongena*	PI 179997	S	S	3.34	4.75	15.88	5
*S. melongena*	PI 181896	MS	S	1.91	6.60	12.54	5
*S. melongena*	PI 181963	S	S	3.99	4.04	15.95	5
*S. melongena*	PI 193599	S	MS	1.84	6.45	11.64	5
*S. melongena*	PI 199516	S	S	1.74	8.76	14.23	2
*S. melongena*	PI 200881	S	S	3.82	5.85	22.10	1
*S. melongena*	PI 204731	S	S	2.73	7.55	18.06	5
*S. melongena*	PI 213193	S	S	1.07	9.21	9.75	5
*S. melongena*	PI 217962	S	S	3.26	4.75	15.36	4
*S. melongena*	PI 223844	S	S	2.89	5.27	14.82	2
*S. melongena*	PI 230333	S	S	7.15	3.62	25.64	4
*S. melongena*	PI 230334	S	S	6.84	3.10	21.22	5
*S. melongena*	PI 230335	S	S	7.43	3.52	26.16	1
*S. melongena*	PI 232078	S	S	4.01	4.57	18.15	5
*S. melongena*	PI 232079	S	S	2.36	5.61	13.14	1
*S. melongena*	PI 233916	MS	S	2.37	5.6	13.16	5
*S. melongena*	PI 234632	S	S	0.93	8.72	7.90	5
*S. melongena*	PI 241506	MS	S	2.73	6.08	16.37	2
*S. melongena*	PI 249570	S	S	1.53	5.79	9.96	5
*S. melongena*	PI 256077	S	MS	3.07	5.27	16.05	4
*S. melongena*	PI 263727	S	S	1.95	8.01	12.82	4
*S. melongena*	PI 267104	S	S	3.93	4.86	18.811	5
*S. melongena*	PI 269600	S	S	1.71	7.13	11.56	4
*S. melongena*	PI 276104	S	S	2.22	7.03	15.56	2
*S. melongena*	PI 286099	S	S	5.81	4.52	25.27	5
*S. melongena*	PI 286100	S	S	6.45	4.73	28.12	5
*S. melongena*	PI 290467	S	S	3.34	6.28	20.96	3
*S. melongena*	PI 290469	S	S	2.16	6.99	14.89	4
*S. melongena*	PI 304839	S	S	2.85	6.17	17.51	4
*S. melongena*	PI 320501	S	S	2.18	7.23	15.48	1
*S. melongena*	PI 320504	S	S	4.58	6.58	29.07	1
*S. melongena*	PI 320509	S	S	2.46	6.93	17.07	4
*S. melongena*	PI 349612	S	S	1.44	5.09	7.38	4
*S. melongena*	PI 351129	S	S	5.61	4.70	26.48	4
*S. melongena*	PI 358232	S	S	4.59	4.90	22.35	4
*S. melongena*	PI 358242	S	S	2.16	7.18	14.53	4
*S. melongena*	PI 358244	S	S	5.36	4.70	24.73	4
*S. melongena*	PI 368822	S	S	3.18	5.81	18.22	1
*S. linnaeanum*	PI 388846	MR	S	1.03	2.38	2.46	3
*S. linnaeanum*	PI 388847	MR	S	1.07	1.89	2.01	3
*S. melongena*	PI 391646	S	S	5.33	8.20	25.77	1
*S. melongena*	PI 413782	S	R	0.79	1.48	1.16	3
*S. melongena*	PI 413783	MR	R	0.46	4.37	2.02	3
*S. melongena*	PI 413784	R	R	0.69	5.85	4.05	3
*S. melongena*	PI 419198	S	MS	5.63	4.38	24.25	1
*S. linnaeanum*	PI 420415	S	S	1.06	2.07	2.14	3
*S. gilo*	PI 441908	R	MR	0.83	5.15	4.27	3
*S. melongena*	PI 452122	S	S	5.79	4.08	23.75	1
*S. melongena*	PI 452123	S	S	1.29	9.60	12.16	1
*S. melongena*	PI 462370	S	S	1.15	10.94	12.02	5
*S. melongena*	PI 470273	S	S	3.30	4.88	15.86	3
*S. melongena*	PI 478390	S	S	0.75	9.54	7.08	4
*S. melongena*	PI 491192	S	S	4.95	4.46	22.06	1
*S. incanum*	PI 500922	MS	R	1.03	2.56	2.63	3
*S. melongena*	PI 560903	S	S	0.95	8.70	8.15	1
*S. melongena*	PI 561139	S	S	2.94	5.58	16.15	1
*S. melongena*	PI 561140	S	S	3.48	4.65	16.2063	4
*S. melongena*	PI 593748	S	S	2.65	5.77	15.18	1
*S. melongena*	PI 593806	S	S	3.79	4.20	15.86	1
*S. melongena*	PI 593885	S	S	1.12	6.00	6.57	2
*S. melongena*	PI 595220	S	S	2.70	4.56	12.54	5
*S. melongena*	PI 600912	S	S	4.58	3.35	15.3	2
*S. melongena*	PI 606714	S	S	2.40	5.60	13.26	3
*S. melongena*	PI 639121	S	S	1.96	7.30	14.02	1
*S. melongena*	PI 639122	S	S	1.60	6.70	10.77	5

a Mean disease rating across all experimental replicates for each isolate, 12889 and OP97.

b Mean fruit parameters for ratio (fruit length:fruit width), length (cm), and width (cm).

c STRUCTURE genetic cluster assignment based on 22 SSR markers.

**Figure 2:**
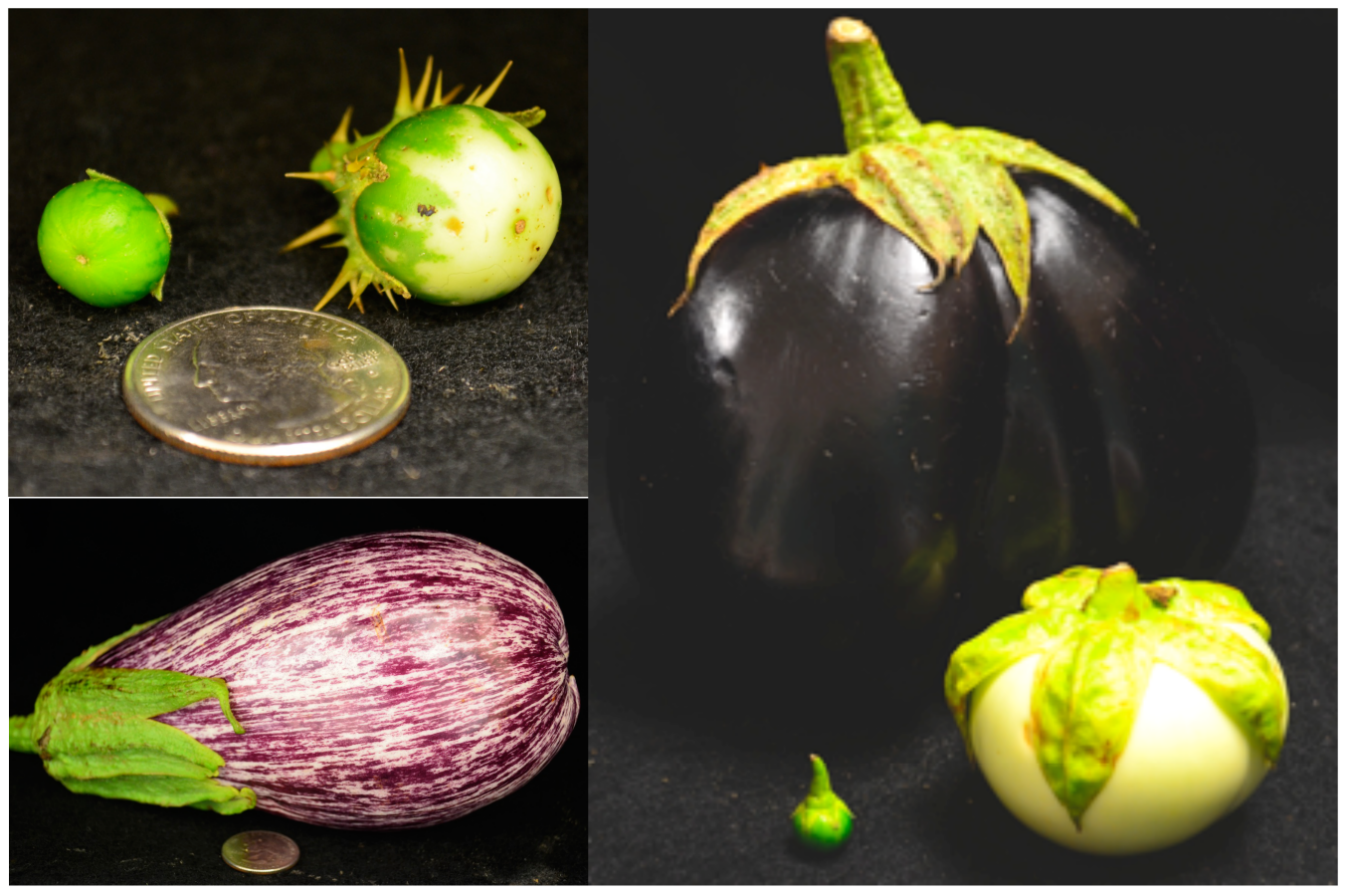
Fruit size and shape differences between eggplants. *Solanum incanum* (left) and *S. linnaeanum* (right) fruit (A), *S. melongena* fruit (B) and *S. linnaeanum* and *S. melongena* fruit varying in shape, size and color (C). U.S. quarter used for size reference.

Fruit shape and size varied considerably in the population (Fig 2, Table 2). *S. melongena* accessions had fruit shape ratios ranging from 1 (round) to 8 (elongate). The wild species evaluated (*S. linnaeanum, S. gilo,* and *S. incanum*) had a fruit shape ratio of approximately 1 (round) with fruit ≤3 cm (Table 2). *Solanum melongena* line PI 413783 had the lowest fruit shape ratio (0.46) and line MM 522 had the highest fruit shape ratio (8).

When evaluated by country, *S. melongena* fruits from Japan had the highest length:width ratio indicating fruits were slender and elongated. Fruits from Thailand had the lowest fruit shape ratio, indicating fruits were more round. Fruit length and width also varied greatly between countries. Fruits from China were the widest and Japan the narrowest. Fruits from Japan were also the longest and fruits from Thailand were the shortest (Table 3).

**Table 3:** *Solanum* spp. fruit shape, width and length variation between countries of origin.

	Fruit					
Category^a^	Shape^b^		Width (cm)^c^		Length (cm)^d^	
**China**	3.0	cd	7.4	A	15.9	de
**France**	3.8	b	5.8	Cd	18.9	b
**Iran**	2.3	de	6.9	Ab	15.4	de
**Italy**	3.5	bc	6.8	Abc	18.0	bcd
**Japan**	6.8	a	3.7	E	24.9	a
**Macedonia**	3.8	b	5.6	Cd	20.0	b
**S. Africa**	2.4	de	6.5	Abc	13.7	ef
**Spain**	3.1	c	6.0	Bc	15.9	d
**Thailand**	2.2	d	5.6	Cd	11.8	f
**Turkey**	3.9	b	5.1	D	18.4	bc
**USA**	3.6	bc	5.7	Cd	16.9	cd

a Categories with less than five individuals representing a country were not included in analyses.

b Mean fruit shape calculated as the ratio of fruit length to fruit width.

c Mean fruit width at midpoint measured in cm.

d Mean fruit length from peduncle to blossom end measured in cm.

### Diversity of SSR Loci in the Eggplant Germplasm Collection

The 192 primers evaluated yielded 22 polymorphic markers that were used for characterizing and evaluating genetic diversity of the eggplant collection (Table 4). A total of 83 alleles were detected among the 22 SSRs, ranging from 2 to 7 alleles per locus with an average allele diversity of 3.8 alleles per locus. The mean genetic diversity index of the collection was 0.49 ranging from 0.03 (T0633) to 0.76 (CSM31) (Table 4). The mean polymorphism information content (PIC) value was 0.42 and individual markers ranged from 0.03 to 0.71 for the population. The highest PIC value was 0.35 in PI 290467 and the lowest PIC value was 0.085 in Grif 1276. Genetic diversity was equally distributed within continents (0.46–0.51), and pairwise Fsts indicated low to moderate genetic differentiation between continents (0.00–0.11) (Table 5). Genetic diversity within countries was similar (0.35–0.48) (Table 6), and pairwise Fst values suggested low (0.00) to great (0.17) genetic differentiation among countries (Table 7). Pairwise Fsts for disease resistance to isolates 12889 and OP97 showed little (0.00) to very great (0.52) genetic differentiation between categories (Table 8). MR/R phenotypes only had high and significant (*P* = 0.05) genetic differentiation with S phenotypes for isolate OP97. Significant genetic differentiation was also detected between the MS and the S category for isolate 12889. No significant genetic differentiation was found between the R/MR category and the MS categories for either *Phytophthora* isolate (Table 8–9). Genetic diversity of fruit shape categories was moderate to high (0.43–0.52) (*data not shown*). Pairwise Fst differentiation between fruit shape categories was low (0.00) to moderate (0.1) (Table 9). Individuals with an elongate fruit shape were significantly differentiated from those with a round or oval fruit shape. Significant differentiation was also detected between round shaped individuals and semi-elongated individuals (Table 9).

**Table 4:** Polymorphic primers evaluated against 99 eggplant lines.

SSR	Forward sequence	Reverse sequence	Allele^a^	Genetic Diversity	PIC^b^	Source
BM61461	CTCATTACCACTTCATACAAAACAG	TGCAGTAGGTGTTGCTACGG	6	0.18	0.18	SolCAP
GPMS203	CACCAACACATCTTTTTCAACC	ATAATAGTGGTTGCGGCGAC	4	0.24	0.23	SolCAP
CB164833	CGGGCAGGTGCTATTATAAAAC	CGGCCGAGGTACAAGCC	3	0.57	0.49	SolCAP
T0633	GATGGGCTATGCTTGCTGTT	ACATCCCCAATGTTGTTGTG	2	0.03	0.03	SolCAP
CA516334	ACCCACCTTCATCAACAACC	ATTTGTGGCTTTTCGAAACG	6	0.61	0.55	SolCAP
GPMS178	GATTTTTGACATGTCACATTCATG	AACGTTGAAAAATAAAGTAAGCAAG	5	0.73	0.69	SolCAP
GP1102	GAACCCTTCATTCCTGTATGT	TTTGCCCGCATTATGTAAATC	2	0.45	0.35	SolCAP
C2_At5g34850	AGTGAAGTGGCTACATCCAAAATCTC	GAACAAAACATGCCCTACTGTAGGAA	7	0.60	0.51	SolCAP
C2_At1g69210	AGCTCTATTCATTTAAAACTAGTCCTCAT	TCTTTTCTTGTATTGGCGGCTAAATTC	2	0.50	0.37	SolCAP
AF348141	CCTTACGGGGAAAACCTAGC	CCATACGGACGTTGTCCTCT	5	0.68	0.62	NCBI
CAMS362	CCCCTTCTGACCTTGATTGA	TATGCCCCTCCTGTGATAGC	4	0.48	0.42	Minamiyama 2007
GO496268.1	CGTTGCCTGTTTACCAACCT	CCTTCTTCTGCACTTCCACA	2	0.48	0.37	NCBI
C2_At5g13200	TATGGGTCCGCCTGCAGTTCCAAC	AAGTTTTCCCCATGCCGCTTCTGT	3	0.11	0.10	SolCAP
C2_At1g32410	TGTTAGTGTCTGGAGGGATTGTATTG	AGATTCGGTGTAGAGACTGGAAGTATC	4	0.64	0.57	SolCAP
CSM7F	CGACGATCACCTTGATAACG	CCTTAAATGCAGAGTTTCCAAAG	2	0.50	0.37	Hurtado 2012
CSM27	TGTTTGGAGGTGAGGGAAAG	TCCAACTCACCGGAAAAATC	3	0.57	0.50	Hurtado 2012
CSM30	CACTGTTCCTGGTTGCTGTG	TTTAGCTTTAGCCCATCTACCG	3	0.50	0.40	Hurtado 2012
CSM31	CAACCGATATGCTCAGATGC	CGGGTATGGTCATGTTTTGC	6	0.76	0.71	Hurtado 2012
CSM43	ATTTTAACCCCGGGAAAATG	ACCGCTTCTAGGTTTTGCAC	4	0.62	0.55	Hurtado 2012
CSM44	CGTCGTTGTAACCCATCATC	TTGCCAAATTCCTTGTGTTC	3	0.46	0.36	Hurtado 2012
CSM54	ATGTGCCTCCATTCTGCAAG	TGGGTGGGATGCTGAGTAAG	3	0.44	0.37	Hurtado 2012
CSM73	TTCAACATAGCCTGGACCATT	AATGCAGGGTTTGGACTTCA	4	0.63	0.56	Hurtado 2012

a Number of unique alleles detected in the population.

b Polymorphism information content for each marker.

**Table 5:** Genetic differentiation (pairwise Fst) estimates of SSRs for *S. melongena* grouped by continent.

	Fst^b^			
Category^a^	Africa	Asia	Europe	N. America
**Asia**	0.00	-		
**Europe**	0.04*	0.02	-	
**N. America**	0.04*	0.00	0.03*	-
**S. America**	0.03	0.11*	0.05	0.06*

a Categories with less than four lines were excluded from analyses and are not shown.

b Average values for SSRs are presented; * indicates value was outside the 2.5% and 97.5% confidence intervals at 1000 bootstraps.

**Table 6:** Genetic diversity estimates for SSRs for *S. melongena* grouped by continent and country of origin.

	Diversity estimates^b^		
Category^a^	AlleleNo	G_D_	PIC
**Africa**	2.71	0.51	0.43
**Asia**	3.10	0.48	0.41
**Europe**	2.81	0.48	0.41
**N. America**	3.10	0.50	0.44
**S. America**	2.67	0.46	0.39
**China**	2.18	0.39	0.32
**France**	2.14	0.38	0.31
**Iran**	2.23	0.42	0.35
**Japan**	1.91	0.35	0.28
**Macedonia**	2.18	0.40	0.33
**South Africa**	2.14	0.41	0.34
**Spain**	2.50	0.42	0.36
**Thailand**	2.36	0.42	0.36
**Turkey**	2.36	0.40	0.34
**USA**	2.77	0.48	0.42

a Categories with less than four lines were excluded from analyses and are not shown.

b Mean values are presented for the average number of alleles (AlleleNo), genetic diversity (G_D_) and the polymorphism information content (PIC).

**Table 7:** Genetic differentiation (pairwise Fst) estimates of SSRs for *S. melongena* grouped by country.

	Fst^b^								
Category^a^	China	France	Iran	Japan	Macedonia	S. Africa	Spain	Thailand	Turkey
**France**	0.00	-							
**Iran**	0.00	0.04	-						
**Japan**	0.01	0.04	0.05	-					
**Macedonia**	0.06	0.13*	0.09*	0.08	-				
**S. Africa**	0.04	0.08	0.01	0.07	0.05	-			
**Spain**	0.06	0.04	0.04	0.13*	0.13*	0.09*	-		
**Thailand**	0.05	0.04	0.07*	0.15*	0.10*	0.04	0.01	-	
**Turkey**	0.04	0.09	0.06	0.15*	0.03	0.05	0.09	0.04	-
**USA**	0.05	0.10*	0.04	0.17*	0.10*	0.05	0.04*	0.07*	0.05

a Categories with less than four lines were excluded from analyses and are not shown.

b Average values for SSRs are presented; * indicates value was outside the 2.5% and 97.5% confidence intervals at 1000 bootstraps.

**Table 8:** Genetic differentiation (pairwise Fst) estimates of SSRs for eggplant germplasm grouped by disease resistance.

	Fst^b^		
Category^a^	R/MR	MS	S
**R/MR**	-	0.19	0.52*
**MS**	0.01	-	0.15
**S**	0.00	0.03*	-

a 12889 appears below the diagonal and OP97 values are above the diagonal; MS =  moderately susceptible, R/MR  =  resistant/moderately resistant, S = susceptible.

b Average values for SSRs are presented; * indicates value was outside the 95% confidence interval at 1000 bootstraps.

**Table 9:** Genetic differentiation (pairwise Fst) estimates of SSRs for *S. melongena* germplasm grouped by fruit shape.

	Fst^b^		
Category^a^	Elongate	Oval	Round
**Oval**	0.10*		
**Round**	0.06*	0.00	
**Semi-Elongate**	0.04	0.02	0.03*

a Fruit shape category based on the ratio of mean length:mean width for each line.

b Average values for SSRs are presented; * indicates value was outside the 2.5% and 97.5% confidence interval at 1000 bootstraps.

### Population Structure Analysis

**Figure 3:**
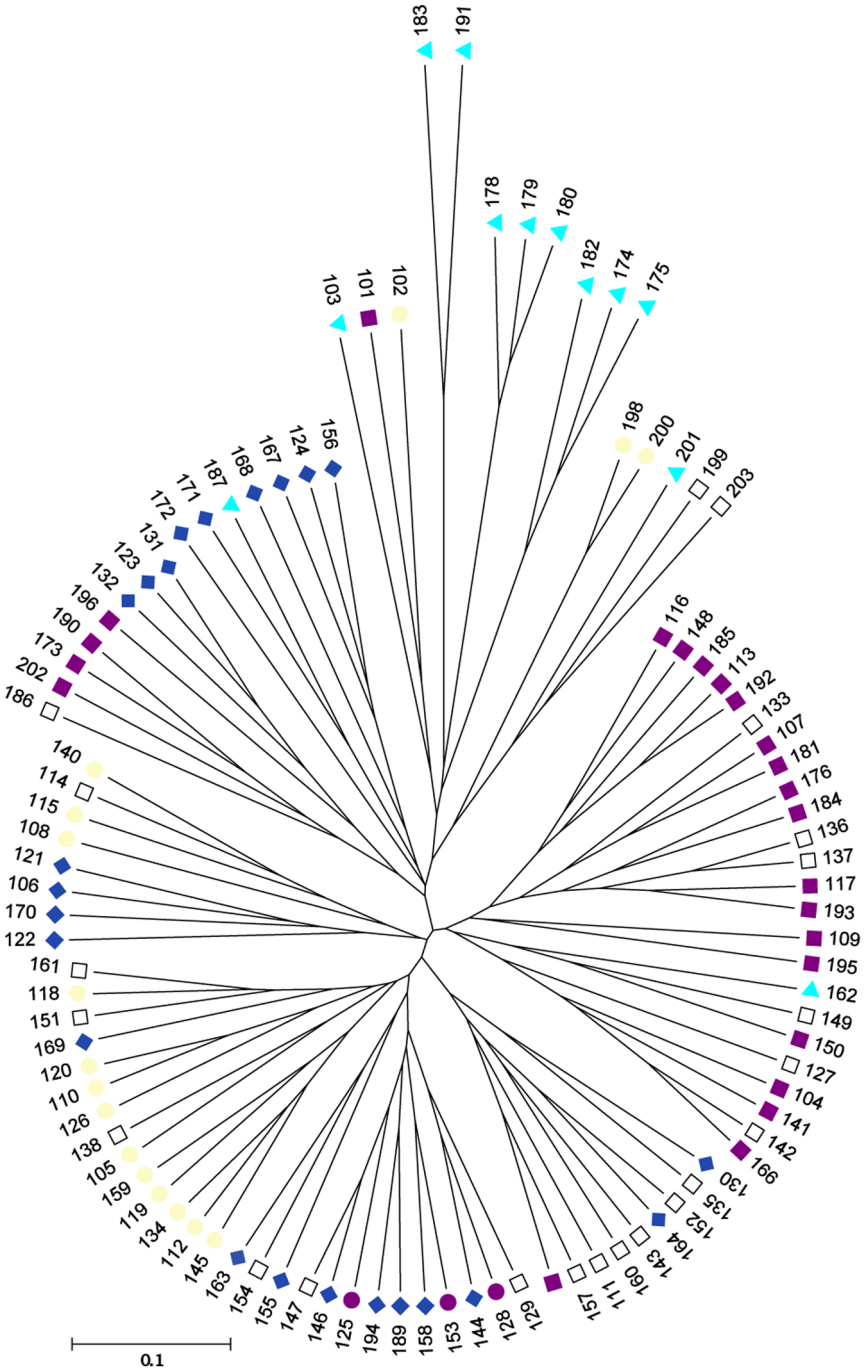
UPGMA genetic distance matrix differences between eggplant lines. Lines are colored based on their STRUCTURE inferred subpopulations. Cluster 1 individuals are denoted by purple squares, Cluster 2 individuals are black-outlined light yellow circles, Cluster 3 individuals are sky blue counterclockwise triangles, Cluster 4 individuals are steel blue diamonds, and admixed individuals are open squares.

**Figure 4:**
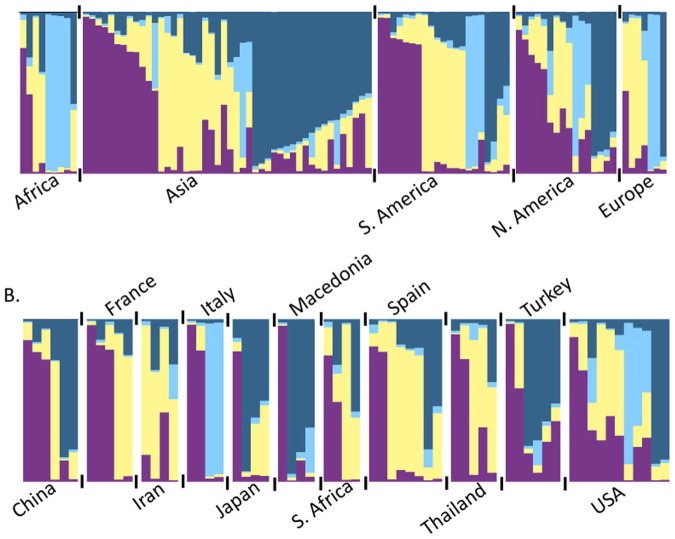
Population structure grouped by country (A) and continent (B) of origin for eggplant (*S. melongena*) germplasm. Cluster 1 (purple), Cluster 2 (light yellow), Cluster 3 (sky blue) and Cluster 4 (dark blue). A white space and black tick marks separate subgroups of individuals. (A) Population structure grouped by country of origin for the *S. melongena* germplasm. Only countries represented by four or more individuals were included (A).

**Figure 5:**
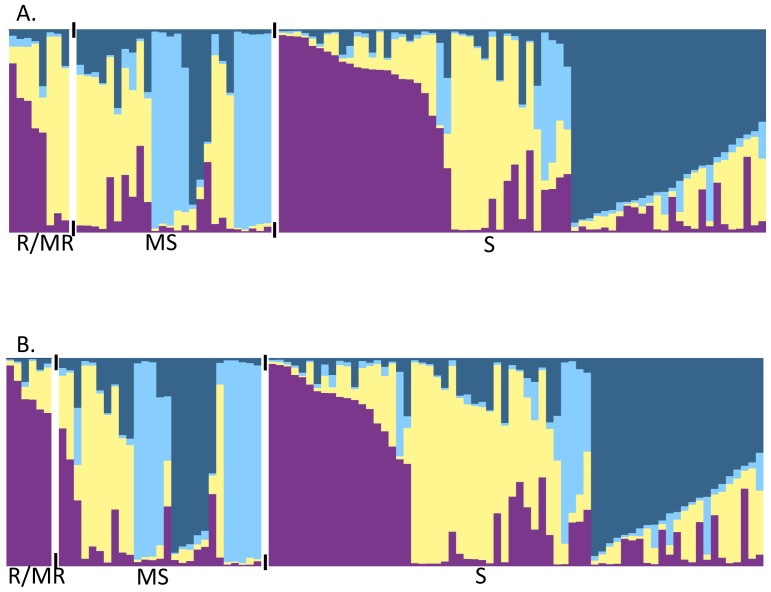
Population structure grouped by disease resistance to isolate 12889 (A) and OP97 (B). Individuals were grouped into a resistant and moderately resistant category (R/MR), a moderately susceptible category (MS), and a susceptible category (S) based on their mean disease ratings. Cluster 1 (purple), Cluster 2 (light yellow), Cluster 3 (sky blue) and Cluster 4 (dark blue). A white space and black tick marks separate subgroups of individuals.

**Figure 6:**
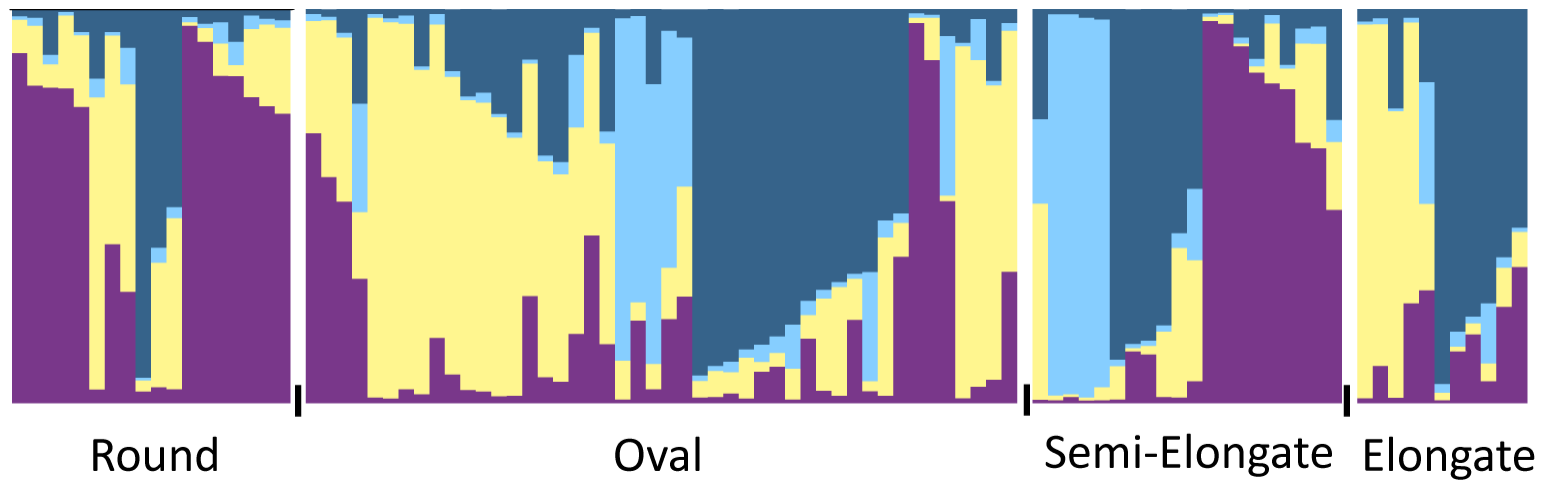
Population structure grouped by *S. melongena* fruit shape. Cluster 1 (purple), Cluster 2 (light yellow), Cluster 3 (sky blue) and Cluster 4 (dark blue). A white space and black tick marks separate subgroups of individuals.

Population structure of the 99 accessions was estimated using the STRUCTURE software and the 22 polymorphic SSRs. Accessions were grouped into four genetic clusters (Ln = - 3381.8). *S. linnaeanum, S. gilo* and *S. incanum* accessions were placed into genetic Cluster 3, while *S. melongena* individuals were distributed through each of the clusters (Table 2). Seventy-eight individuals could be assigned to a single cluster based on membership, while the remaining 21 individuals could not be assigned and were classified as admixed. When compared with the UPGMA tree, STRUCTURE-inferred clusters largely overlapped with the grouping of branches based on genetic distance (Fig 3). Relationships between the inferred clusters according to the UPGMA tree indicated that Cluster 3 was more differentiated from Clusters 1, 2 and 4, and that Clusters 1 and 4 and Clusters 2 and 4 were less differentiated from each other. Pairwise Fsts between clusters were significant and ranged from 0.08 to 0.17, indicating 8–17% of the variation was explained by genetic differences between clusters. Cluster 1 had moderate differentiation from Clusters 2, 3 and 4. Cluster 2 had great differentiation from Cluster 3, and Cluster 4 had moderate differentiation from Clusters 2 and 3 following the guidelines suggested by Hartl and Clark [55]. Population structure was detected when individuals were grouped by continent of origin (Fig.4A), country of origin (Fig. 4B), species, fruit shape (Fig. 5) and disease resistance to isolates 12889 and OP97 (Fig. 6), as some clusters were more frequent than others in each grouping (Fig. 4–6). Cluster 3 individuals were not represented in Asia, and Cluster 4 individuals were not represented in Africa (Fig 4). For both isolates, individuals from Cluster 4 were not represented in the MR/R categories for either isolate, had low representation in the MS category, and were highly represented in the S category. Cluster 3 individuals were highly represented in both the R/MR and S categories, but not the MS for both isolates (Fig. 6). When grouped by fruit shape (round, oval, semi-elongate and elongate), Cluster 1 was under represented in the oval and elongate fruit shape categories. Clusters 2 and 4 both had low representation in the round category. Cluster 3 was not represented in round or elongated individuals and had minor representation in the oval shape category.

## Discussion

This study investigated Phytophthora fruit rot resistance, fruit shape, population structure and genetic diversity in a worldwide collection of eggplant. The overall estimate of genetic diversity of the collection was moderate (0.5) in our study, similar to a recent report on eggplant diversity, though this is likely to be an underestimation due to limited sampling [3]. Bayesian clustering, using SSR markers, identified four genetic clusters in the eggplant collection. Most individuals belonged predominantly to one of the four clusters, while 20% were admixed according to the inferred clustering. Admixture, an indicator of migration or interbreeding between genetic clusters, was moderate in our population. Inferred genetic clusters did not directly correspond with the predefined categories of continent, country, fruit shape or Phytophthora fruit rot resistance, though some clusters did appear more frequently in one category compared to another.

On eggplant, fruit rot is the most common symptom of *P. capsici* seen in the field [23]. Symptoms start as small water soaked lesions, turning brown and eventually covering the whole fruit. Advanced symptoms can include complete rotting of the fruit and visible mycelia on the external surface of the fruit [23]. Isolate-specific interactions and partial fruit rot resistance have been identified in other solanaceous species (tomatoes and peppers) suggesting a multigenic host response, but no studies have looked at Phytophthora fruit rot in eggplant [24] (Granke et al. unpublished). In our study, the eggplant accessions evaluated demonstrated partial and isolate-specific resistance to Phytophthora fruit rot. Most lines evaluated were completely susceptible to both isolates (∼90%). Several eggplant accessions displayed isolate-specific resistance; these individuals were placed into genetic Clusters 2 and 3, and were from S. America, Asia, Africa and Europe. Two of these geographic regions are known centers of eggplant diversity, and likely harbor additional sources of resistance [2]–[5]. Only one of the 99 lines evaluated, a Cluster 3 landrace eggplant collected in Burkina Faso in the early 1900s, had complete resistance to both isolates evaluated. This accession also showed high levels of genetic similarity to the wild eggplant relatives evaluated, *S. incanum, S. gilo*, and *S. linnaeanum*. While further evaluation with more isolates is necessary, PI 413784 appears to be a promising source of host resistance to Phytophthora fruit rot in eggplant.

When categorized by disease resistance (S, MS, MR, R) for each isolate, there was significant genetic differentiation among eggplant genotypes infected with isolates OP97 or 12889. Individuals that were R and MR to isolate OP97 were significantly differentiated from individuals that were S. Only S individuals were significantly differentiated from the MS individuals when inoculated with isolate 12889. These results emphasize the importance of utilizing different *P. capsici* isolates when breeding for resistance. The three wild relatives, *S. linnaeanum, S. gilo*, and *S. incanum*, showed partial or isolate-specific resistance to the two isolates evaluated in this study.

When grouped by species, *S. linnaeanum*, *S. gilo*, and *S. incanum* individuals evaluated were predominantly in genetic Cluster 3. *Solanum incanum* has long been part of the eggplant complex and ancestral individuals are thought to be one of the progenitors of modern eggplant [43], [44]. *Solanum linnaeanum* is a related species and has only recently been included as a possible progenitor of the modern eggplant with limited crossability [5], [8]. Genetic Cluster 3 individuals were also detected in the *S. melongena* category, supporting gene movement between *S. melongena* and its wild relatives, *S. incanum* and *S. linnaeanum*. These *S. melongena* individuals may have been misclassified, but are more likely the result of introgression since the wild species were small fruited and prickly.

Cultivated eggplant, similar to pepper and tomato, is a phenotypically diverse species with varying levels of genotypic diversity [42], [44], [46], [57]. *Solanum melongena* fruit shape, size and color is a byproduct of domestication, selection, and breeding for specific market classes. Phenotypic evaluation of eggplant fruit shape varied greatly among the *S. melongena* accessions evaluated, while the wild species, *S. incanum, S. gilo, and S. linnaeanum*, had no variation in fruit shape. Maintaining market class variation may be difficult when incorporating traits like fruit rot resistance, which was most often observed in accessions with a limited size range.

Significant differences in this population were observed in fruit shape, length and width among eggplant lines when grouped by country of origin, representing different market classes, in this study. Since eggplant has market classes particular to geographic areas, it was expected that population structure categorized by fruit shapes and country of origin would correspond with the inferred genetic clusters. Significant differentiation was seen between *S. melongena* individuals with elongated fruit shapes and those with round and oval fruit shapes. Individuals with a round fruit shape were also significantly differentiated from semi-elongate fruit shape individuals. These results are consistent with limited breeding among market classes. However, inferred population structure did not correspond with the fruit categories and this may be the result of limited sampling in each geographic location. While only genetic Cluster 3 was not represented in the round or elongate shape category, all other clusters were represented by at least one individual in each category.

When grouped by country and continent, significant population structure and moderate genetic diversity was evident among the categories evaluated. The highest levels of genetic diversity were seen within the continents of Africa and N. America. The highest level of genetic diversity for countries was in the USA. The increased genetic diversity in Africa is likely due to the prevalence and intercrossing of wild and related species, as Africa is the center of origin for several eggplant species. The increased genetic diversity in N. America and the USA may be the result of breeding programs integrating wild relatives and varieties from around the world. The diversity could also be from the movement of Asian and European varieties into the U.S., which may be marketed under different names. Overall differentiation among countries was similar to the differentiation among continents, and future core collections should include individuals from areas with high genetic diversity and genetic differentiation. In particular, genotypes from China were not significantly differentiated from any other country, while genotypes from Thailand, Japan, Spain, Macedonia and the U.S. were frequently significantly differentiated from other countries. Similarly, Asia was not significantly differentiated from populations from Europe, Africa and N. America, while N. America, Europe and Africa were all significantly differentiated from each other. Asia, as a center of diversity and domestication, and in particular genotypes from China, may be more akin to the ancestral population from which these other pools were derived, making them more similar and less differentiated from other eggplants.

Cultivated eggplant, compared to other solanaceous species, is an understudied crop with worldwide importance. This study provides an overview of the population structure, genetic diversity and Phytophthora fruit rot resistance of a geographically diverse set of eggplant. The estimates of genetic diversity and the four genetic clusters found in this study are likely to be lower than actual genetic diversity and structure of eggplant due to limited sampling and molecular markers. A previous study using a subset of SSRs in a smaller collection of eggplant was able to identify more allelic variation at each locus [3]. While population structure was significant for disease resistance, fruit shape, continent and country, the genetic clusters did not completely correspond with these predefined categories in our study, which may be due, in part, to unequal samples in each category. Future studies involving eggplant diversity, disease resistance and other agronomic traits should aim to include individuals from around the world for maximum diversity, and will need to consider the effect of population structure on marker-trait associations.
